# Therapeutic Effect of Insulin in Reducing Critical Illness; Polyneuropathy and Myopathy in the Pediatric Intensive Care Unit 

**Published:** 2012

**Authors:** Nemat BILAN, Shahram SADEGVAND, Shirin RANJBAR

**Affiliations:** 1 Professor of Pediatric Pulmonology, Tuberculosis and Lung Disease Research Center, Tabriz University of Medical Sciences, Tabriz, Iran; 2 Pediatrician, Tabriz University of Medical Sciences, Tabriz, Iran; 3 Medical student, Tabriz University of Medical Sciences, Tabriz, Iran

**Keywords:** CIPNM, Insulin, Child

## Abstract

**Objective:**

Hyperglycemia may occur in the patients affected by any kind of critical illness.

This complication makes an adverse effect on the clinical outcome of these patients by causing polyneuropathy and myopathy. It has been recently shown that treatment of hyperglycemia with insulin administration significantly reduces the prevalence of critical illness polyneuropathy and myopathy (CIPNM) and on the other hand reduces the demand for long-term mechanical ventilation in the patients admitted to the ICU for more than 1 week. The aim of this study was to determine the therapeutic effect of insulin in reducing the incidence of CIPNM in the pediatric intensive care unit (PICU).

**Materials & Methods:**

In this study, we recruited 30 patients admitted to the PICU of Tabriz Pediatric Hospital. The incidence of CIPNM following hyperglycemia was evaluated in these patients. The patients were categorized into two groups. In the case group, blood sugar was controlled in the range of 140-180mg/dl by administration of 0.05 unit per kilogram body weight of insulin as drip protocol in an hour and in the control group, placebo was used. Consequently, the incidence of CIPNM, duration of PICU and duration of mechanical ventilation were compared between the two groups.

**Results:**

The incidence of CIPNM and duration of PICU stay and mechanical ventilation were significantly reduced in the patients treated with insulin compared to the control group.

**Conclusion:**

This study shows that blood sugar control decreases the incidence of CIPNM.

## Introduction

Patients who have been admitted to the intensive care unit for more than five days because of sepsis and multiorgan dysfunction and critical illness polyneuropathy are exposed to a high risk of morbidity and mortality (1). Polyneuropathy and myopathy are considered as the serious complications of critical illness. Sepsis, multiorgan dysfunction and hyperglycemia in admitted patients aggravate the polyneuropathy and myopathy and the attempt to prevent them has a significant role in reducing polyneuropathy and myopathy. Hyperglycemia developed by critical illness may continue for several days or may resolve in a few days (2, 3). In the patients affected by sepsis and multiorgan dysfunction, the peripheral nervous system is one of the systems that may be involved. The result of this diffused peripheral neuropathy has been nominated as critical illness polyneuropathy (2). Nerve conduction studies in the patients affected by sepsis and multiorgan dysfunction have demonstrated sensory and motor polyneuropathy in 70 percent of the patients (4). These problems are hidden in most of the patients, but in some cases they may cause severe weakness in the extremities and the trunk (5).

In a 24-month observational study, 57 patients who were admitted to the intensive care unit of Tabriz children hospital and were mechanically ventilated for at least one week were recruited. Based on clinical and electrodiagnostic findings, critical illness polyneuropathy and myopathy (CIPNM) was diagnosed in 13 patients (22.8%). In this study, hyperglycemia as one of the CIPNM causing factors was analyzed. Hyperglycemia may occur in patients affected by any critical illness and may cause an adverse effect in the outcome of these patients. It has been shown recently that treatment with insulin significantly reduces the prevalence of CIPNM. On the other hand, it reduces the demand for long-term ventilation in patients who are admitted to the ICU for more than 1 week (6). Therefore, with regard to the limited studies investigating the therapeutic effects of insulin in reducing CIPNM in children and also to prevent acquired paresis unrelated to the central nervous system diseases, this study assessed the important role of insulin therapy in CIPNM.

## Materials & Methods

We conducted an interventional study (a clinical trial) and evaluated 30 patients admitted to the PICU of Tabriz Pediatric Educational Therapeutic Center from August 2011 to February 2012. In 18 months, 30 patients who were admitted to the PICU and had received mechanical ventilation for at least 1 week and had developed hyperglycemia (BS>180 mg/dl) during their hospitalization were recruited. The effect of hyperglycemia on the incidence of CIPNM was evaluated. 

Patients with spinal lesions, Guillain-Barre syndrome, myasthenia gravis, hypophosphatemia, patients who developed nervous system disorders and complications during their hospitalization, patients with confirmed neuromuscular diseases that caused weakness and paralysis, patients with a blood sugar level higher than 250 mg/dl and patients with known metabolic disorders were excluded from the study.

Patients who were enrolled into the study were categorized into two groups. In the first group insulin was administrated to 15 patients who were under mechanical ventilation in order to maintain blood sugar in the range of 140-180 mg/dl. In the other group (the other15 patients), placebo was used instead. These patients were continuously examined during their hospitalization and the status of their deep tendon reflexes and weakness was evaluated. In each patient suspicious of polyneuropathy and myopathy, EMG and NCV was conducted at earliest convenience after weaning from the ventilator. NCV of motor nerves (tibial, peroneal and median) and sensory nerves (soral, median, f wave of tibial and ulnar nerves) and EMG of the proximal and distal muscles of the lower and upper extremities were performed. On the basis of the results of EMG and NCV in the two abovementioned groups, the intensity of polyneuropathy and myopathy was classified into three categories: 

1.Sensory loss in the lower limbs (mild) 

2. Sensory-motor loss in the lower limbs and sensory loss in the upper limb (moderate) 3. Sensory-motor loss in the lower and upper limbs (severe).

The treatment protocol was continuous administration of 0.05 unit per kilogram of body weight insulin as drip infusion in an hour and serial control of blood sugar every hour with the aim of maintenance of blood sugar in the range of 140-180mg/dl. When the blood glucose level fell below the targeted range, insulin therapy was stopped.

This study was approved by the Ethics Committee of Tabriz University of Medical Sciences and the Iranian registry for clinical trials (IRCT). The clinical trial number was 201108117295 N1. Written consent was obtained from the parents and patient information was kept confidential.

Data were analyzed using SPSS 13 for Windows (SPSS Inc., Chicago, Illinois, USA). The quantitative variables (mean days of PICU stay and the mean time of mechanical ventilation) were compared using the Mann-Whitney U test. The quantitative variables were compared using the chi-square test. A p value ≥ 0.05 was considered statistically significant. The data were presented as mean ± SD, frequency and percentage values.

## Results

The patients had an age range between 1 month to 12 years. Of the 30 patients recruited in this study, 15 patients were treated with insulin and placebo was used for the other 15 patients. Of the 15 patients treated with insulin, CIPNM was diagnosed in six patients (40%) and nine patients did not show CIPNM (60%). Of the 15 patients in the control group, CIPNM was diagnosed in 11 cases (73.3%) and four cases did not have CIPNM (26.7%). There was a statistically significant intergroup difference in the incidence of CIPNM (p=0.023). 

The mean duration of ICU stay was 15.13 days in the patients treated with insulin and 19.73 days in the control group indicating a statistically significant difference in the duration of ICU stay between the two groups (p>0.001). In the insulin administered group, the duration of mechanical ventilation was 12.87±2.13 days and in the control group this figure was 16.33±2.71 days. There was a significant difference in the duration of mechanical ventilation between the two groups (p>0.001).

The mean PRISM (Pediatric Risk of Mortality) score was 15.7±3.28 in the group treated with insulin and 16.93±2.78 in the control group. There was no significant difference statistically in the PRISM score between the two groups at the time of admission to the PICU (p=0.18).

## Discussion

In the present study, hyperglycemia was evaluated as one of the important factors causing CIPNM in the ICU of Tabriz Pediatric Hospital. Of the 30 evaluated patients, CIPNM was diagnosed in six patients (40%) of the insulin treated group and 11 cases of the control group (73.3%).

In a study conducted by Hermans and colleagues, they concluded that controlling blood sugar by administration of insulin in ICU patients reduced the incidence of CIPNM compared to the control group. In their study, the incidence of CIPNM was 38.9% in the patients treated with insulin and 50.5% in the control group (6).

Besides, in another study they showed that control of blood sugar by daily administration of insulin in the ICU would have a positive effect on the neuromuscular function and would cause reduction in the incidence of CIPNM electrophysiologically (6).

In 2005, in a study conducted by Van den Berghe and colleagues, they showed that administration of insulin in order to prevent hyperglycemia in ICU kept the central and peripheral nervous system safe leading to shortening of ICU stay and consequently earlier remission after discharge (1).

All the studies mentioned above emphasize that there is no doubt that maintenance of blood sugar in the normal range reduces the incidence of CIPNM. The present study is in accordance with these findings. Hermans et al. (6) showed that the patients struggling with critical illnesses in the ICU are exposed to the risk of damage caused by hyperglycemia that is because of the passive offtake of glucose and reduction in the clearance of free oxygen radicals. They also propounded that prevention of hyperglycemia has a simple protective effect on the mitochondrial structure and function of the nervous system.

Maintenance of the normoglycemic state also reduces organ damage by vessel endothelium protection via reduction of nitric acid production. Administration of insulin itself would be beneficial in the patients admitted to the ICU by reducing inflammation and dyslipidemia (6).

In several studies, the target range for blood sugar has been mentioned as 80-110mg/dl and maintenance of blood sugar in this range has been the aim of insulin administration. However, in the two studies conducted by Wiener et al. and Griesdal et al., it was propounded that tight control of blood sugar did not affect mortality and it was associated with a high risk of hypoglycemia (7, 8).

In a study carried out by Mraovic in 2009, a higher mortality rate was detected in patients with a 81-108mg/dl maintained blood sugar level than those with a 140-180mg/dl target range of blood sugar. Hypoglycemia caused by tight control of blood sugar reduces the other profits of blood sugar control (9). In our study, because the patients were children and that children are more sensitive to hypoglycemia, we appointed the range of 140-180mg/dl as the target range for blood sugar in order to prevent complications of hypoglycemia. In our study, there was a significant difference between the control group and the group treated with insulin regarding the duration of ventilator dependency. In the study carried out by De Jonghe and colleagues, they concluded that prevention of acquired neuromuscular injuries in the ICU would shorten the duration of ventilator dependency (10).

Garnacho-Mantero et al conducted a study in 2005 and mentioned that CIPNM significantly increases the duration of mechanical ventilation consequently increasing the ICU stay and hospitalization (11). Hermans and colleagues concluded that controlling blood sugar with insulin in patients admitted to the ICU, definitely with no association to other factors, decreases the risk of long-term mechanical ventilation. It does not seem that this effect occurs by CIPNM reduction alone. It appears that this effect is associated with the dose of insulin administered, because insulin has an anabolic effect on the musculoskeletal system (6).

Parallel to the studies mentioned above, in order to explain the reason of the increase in the duration of mechanical ventilation in this study, it seems that in patients admitted to the ICU, muscle mass ultimately diminishes because of the complete bed rest state. 

This factor causes weakness of the respiratory muscles; therefore, weaning the patients from the ventilator would be difficult. Administration of insulin prevents muscle mass wasting to some extent; subsequently, the duration of mechanical ventilation would be shortened. On the other hand, CIPNM itself causes weakness in the respiratory muscles accompanied by other muscle weakness and it makes weaning problematic in these patients.

In our study, the duration of ICU stay in the patients treated with insulin was significantly shorter than the control group. In the study conducted by Garnacho-Mantero et al., as mentioned before, the duration of ICU stay and consequently the duration of hospitalization in patients with CIPNM has been reported higher than patients without CIPNM (11). It is obvious that in patients with CIPNM and also in patients under longterm mechanical ventilation due to high morbidity more complications, the demand for ICU admission will increase.

Comparison of PRISM score between the two groups at the time of PICU admission was another objective that was evaluated alongside the above mentioned matters. The patients who had a PRISM score below 9 and a PRISM score higher than 30 were excluded from the study. The patients who had a PRISM score below 9 were cases who did not need mechanical ventilation for more than 7 days and were spontaneously excluded from our study. With regard to the point that in patients with a PRISM score higher than 3, factors other than hyperglycemia could lead to polyneuropathy and myopathy, these patients were excluded. The patients with a PRISM score between 10 and 29 were included. There was no significant difference in the PRISM score at the time of PICU admission between the two groups. Since CIPNM is a multifactorial disease, it is recommended to evaluate the other factors separately in other studies.

**In conclusion,** this study shows that controlling blood sugar reduces the incidence of CIPNM, leading to shortening of the duration of mechanical ventilation, ICU stay and finally hospitalization. 

## References

[1] Van den Berghe G (2004). Insulin therapy in critical illness. Can J Diabetes.

[2] Bolton CF, Gilbert JJ, Hahn AF, Sibbald WJ (1984). Polyneuropathy in critically ill patients. J Neurol Neurosurg Psychiatry.

[3] Vondracek P, Bednarik J (2006). Critical and electrophysiological findings and long-term outcomes in pediatric patients with critical illness polyneuropathy. Eur J Pediatr Neurol.

[4] Witt NJ, Zochodne DW, Bolton CF, Grand’Maison F, Wells G, Young GB (1991). Peripheral nerve function in sepsis and multiple organ failure. Chest.

[5] Marino PL (1998). The ICU book.

[6] Hermans G, De Jonghe B, Bruyninckx F, Van den Berghe G (2008). Clinical review: Critical illness polyneuropathy and myopathy. Crit Care.

[7] Wiener RS, Wiener DC, Larson RJ (2008). Benefits and risks of tight glucose control in critically ill adults: a metaanalysis. JAMA.

[8] Griesdale DE, de Souza RJ, van Dam RM, Heyland DK, Cook DJ, Malhotra A (2009). Intensive insulin therapy and mortality among critically ill patients: a meta-analysis including NICE-SUGAR study data. CMAJ.

[9] Mraovic B (2009). Continuous glucose monitoring during intensive insulin therapy. J iabetes Sci Technol.

[10] De Jonghe B, Bastuji-Garin S, Sharshar T, Outina H, Brochard L (2004). Does ICU-acquired paresis lengthen weaning from mechanical ventilation?. Intensive Care Med.

[11] Garnacho-Mantero J, Amaya-Villar R, García-Garmendía JL, Madrazo-Osuna J, Ortiz-Leyba C (2005). Effects of criticall illness polyneuropathy on the withdrawal from mechanical ventilation and the length of stay in septic patients. Crit Care Med.

